# Active versus passive case finding for tuberculosis in marginalised and vulnerable populations in India: comparison of treatment outcomes

**DOI:** 10.1080/16549716.2019.1656451

**Published:** 2019-09-02

**Authors:** Hemant Deepak Shewade, Vivek Gupta, Srinath Satyanarayana, Sunil Kumar, Prabhat Pandey, U. N. Bajpai, Jaya Prasad Tripathy, Soundappan Kathirvel, Sripriya Pandurangan, Subrat Mohanty, Vaibhav Haribhau Ghule, Karuna D. Sagili, Banuru Muralidhara Prasad, Priyanka Singh, Kamlesh Singh, Gurukartick Jayaraman, P. Rajeswaran, Moumita Biswas, Gayadhar Mallick, Ali Jafar Naqvi, Ashwin Kumar Bharadwaj, K. Sathiyanarayanan, Aniruddha Pathak, Nisha Mohan, Raghuram Rao, Ajay M. V. Kumar, Sarabjit Singh Chadha

**Affiliations:** a Centre for Operational Research, International Union Against Tuberculosis and Lung Disease (The Union), Paris, France; b Department of Operational Research, The Union South-East Asia (USEA), New Delhi, India; c Karuna Trust, Bengaluru, India; d Dr RP Centre for Ophthalmic Sciences, All India Institute of Medical Sciences (AIIMS), New Delhi, India; e State TB Cell, Department of Health & Family Welfare, Government of Kerala, Thiruvananthapuram, India; f Department of TB and Communicable Diseases, The Union South-East Asia (USEA), New Delhi, India; g Voluntary Health Association of India (VHAI), New Delhi, India; h Department of Community Medicine and School of Public Health, Post Graduate Institute of Medical Education and Research (PGIMER), Chandigarh, India; i Joint Efforts for Elimination of TB (JEET) Project, Foundation for Innovate New Diagnostics (FIND), New Delhi, India; j MAMTA Health Institute for Mother and Child, New Delhi, India; k Catholic Health Association of India (CHAI), Telangana, India; l Resource Group for Education & Advocacy for Community Health (REACH), Chennai, India; m IIHMR University, Jaipur, India; n Central TB Division, Revised National Tuberculosis Control Programme, Ministry of Health and Family Welfare, Government of India, New Delhi, India; o Yenepoya Medical College, Yenepoya (Deemed to be University), Mangaluru, India; p Infectious Diseases, Foundation for Innovate New Diagnostics (FIND), New Delhi, India

**Keywords:** Tuberculosis/therapy, systematic screening, vulnerable populations, treatment outcome, community-based active case finding

## Abstract

**Background**: Community-based active case finding (ACF) for tuberculosis (TB) implemented among marginalised and vulnerable populations in 285 districts of India resulted in reduction of diagnosis delay and prevalence of catastrophic costs due to TB diagnosis. We were interested to know whether this translated into improved treatment outcomes. Globally, there is limited published literature from marginalised and vulnerable populations on the independent effect of community-based ACF on treatment outcomes when compared to passive case finding (PCF).

**Objectives**: To determine the relative differences in unfavourable treatment outcomes (death, loss-to-follow-up, failure, not evaluated) of ACF and PCF-diagnosed people.

**Methods**: Cohort study involving record reviews and interviews in 18 randomly selected districts. We enrolled all ACF-diagnosed people with new smear-positive pulmonary TB, registered under the national TB programme between March 2016 and February 2017, and an equal number of randomly selected PCF-diagnosed people in the same settings. We used log binomial models to adjust for confounders.

**Results**: Of 572 enrolled, 275 belonged to the ACF and 297 to the PCF group. The proportion of unfavourable outcomes were 10.2% (95% CI: 7.1%, 14.3%) in the ACF and 12.5% (95% CI: 9.2%, 16.7%) in the PCF group (p = 0.468). The association between ACF and unfavourable outcomes remained non-significant after adjusting for confounders available from records [aRR: 0.83 (95% CI: 0.56, 1.21)]. Due to patient non-availability at their residence, interviews were conducted for 465 (81.3%). In the 465 cohort too, there was no association after adjusting for confounders from records and interviews [aRR: 1.05 (95% CI: 0.62, 1.77)].

**Conclusion**: We did not find significant differences in the treatment outcomes. Due to the wide CIs, studies with larger sample sizes are urgently required. Studies are required to understand how to translate the benefits of ACF to improved treatment outcomes.

## Background

Globally, national tuberculosis programmes rely predominantly on passive case finding (PCF) to detect people with tuberculosis (TB). PCF is defined as detecting TB at health facilities among persons who seek medical care on their own. The epidemiological impact of PCF for TB has been inadequate [1–4]. Many people with presumptive TB do not seek care at a health facility and this points towards the need for advocacy, communication and social mobilisation (ACSM) and community-based active case finding (ACF) [5].

ACF is defined as systematic screening for TB applied outside of health facilities [4]. In most high burden settings, ACF can be a powerful and cost-effective tool [6]. Systematic screening of the high-risk groups is one of the components of pillar one (integrated, patient-centred care and prevention) of END TB strategy [7].

A key question while assessing the effectiveness of any TB screening strategy is ‘*Is there a difference in treatment outcomes between people with TB found by screening and those found through PCF?’* [8] When compared to PCF, ACF is expected to detect people with TB at an earlier stage of disease, when they are relatively less sick (early diagnosis). Early diagnosis provides an opportunity to initiate early treatment which in turn is expected to lead to better treatment outcomes. Despite this, a systematic review published in 2013 identified similar treatment success among ACF and PCF-diagnosed people with TB [8]. Studies published after that has presented a mixed picture. An Ethiopian study involving ACF and other interventions like ACSM, LED microscopy, contact tracing, isoniazid preventive therapy and treatment support showed better outcomes after implementation. A Nigerian study involving ACF only showed no difference in outcomes [9,10]. But for a recently published study from Myanmar that reported comparable treatment outcomes, there is limited published literature where the effect of ACF on treatment outcomes (as compared to PCF) has been assessed after adjusting for potential confounders [11]. There are no nationally representative studies where ACF has been assessed for the effect on treatment outcomes in programme settings.

Globally, India has the highest burden of TB [12,13]. High loss to follow-up (pre-treatment and during treatment) has been reported among ACF-diagnosed people with TB when compared to PCF in two separate studies from a district from south India (1999, 2002–03) [14,15]. Unwillingness among patients to start treatment, mild symptoms and dissatisfaction with health system were some of the reasons reported [15].

Since 2010, The Union South-East Asia (based in New Delhi) has been implementing The Global Fund supported Project Axshya (meaning ‘free of TB’) in India to mitigate the impact of TB among vulnerable and marginalized populations through ACSM and ACF [16–18]. The ACF focussed on increasing the detection of people with new smear-positive pulmonary TB. It resulted in detection of a large number of persons with presumptive pulmonary TB and smear-positive pulmonary TB, reduced diagnosis delay, reduced costs due to TB diagnosis and prevalence of catastrophic costs due to TB diagnosis [19–22]. However, we do not know whether this translated into improved TB treatment outcomes.

Therefore, the objective of this study was to determine the relative differences in unfavourable TB treatment outcomes (death, loss-to-follow-up, failure, not evaluated) of ACF and PCF-diagnosed people with new smear-positive pulmonary TB from marginalised and vulnerable populations in India.

## Methods

### Study design

This was a cohort study using primary and secondary data.

### Study setting

#### India’s national TB programme (2016–17)

India is the second most populous country. It is administratively divided into 36 states and union territories, which is further divided into more than 700 districts. As per India’s revised national TB control programme (RNTCP), designated microscopy centers (DMCs-one for 50 000 to 100 000 population) provided sputum microscopy services [23,24]. The basic management units were sub-district level administrative units (called as TB units-one for 250 000 to 500 000 population). TB registers maintained at each TB unit indicated the number of people with TB treated and registered under RNTCP [23,24]. Newly registered people with TB received two months of Isoniazid, Rifampicin, Pyrazinamide and Ethambutol followed by four months of Isoniazid, Rifampicin and Ethambutol [24].

#### ACF under project axshya (2016–17)

In 2016–17, project Axshya covered 285 districts spread across 19 states. Axshya districts and TB units were identified in consultation with the state TB programme. Even within a TB unit, the ACF and ACSM activities were preferentially targeted towards marginalised and vulnerable populations. This included slums, tribal areas, scheduled caste communities (traditionally marginalized and excluded communities as per Constitution of India), areas where a large number of homeless people and people involved in unorganized labour reside, areas where occupational lung diseases are high, areas reported to have high HIV/AIDS burden, areas known to report high incidence of TB (including prisons) and household contacts of smear-positive pulmonary TB patients (see Suppl Annex 1 for details). The district coordinator in Axshya district was supervised by the assistant project manager, supported by the state technical consultant (if available) and project management unit at The Union South-East Asia.

Axshya *SAMVAD* (sensitization and advocacy in marginalised and vulnerable areas of the district) is a community-based ACF strategy. *SAMVAD* in Sanskrit language means ‘conversation’. Trained community volunteers (Axshya *mitras*, meaning friends of Axshya in Hindi) visited households, educated the household members on TB, screened them for TB symptoms and referred people with presumptive pulmonary TB (a referral slip was provided) to the nearest DMC for sputum examination. Sputum collection and transport services were provided in case of ‘failed referrals’ (persons with presumptive TB who did not visit the DMC despite referral) [25]. Project Axshya did not have any treatment support package after TB diagnosis.

The district coordinators provided one-day training to Axshya *mitras* in identifying TB symptoms using the symptomatic verbal screening criteria (more than 2 weeks of cough, evening rise in temperature, loss of appetite, and loss of weight (any one)) and on collection of quality sputum specimen. Axshya *mitras* received activity-based honorarium for every house visit and every sputum collection and transport with in-built quality control mechanisms (sputum positivity rate of 7%) [16]. (see Suppl Annex 1 for details on technical and operational guidelines)

### Study population and sampling

The eligible population were people (≥15 years) with new smear-positive pulmonary TB belonging to marginalised and vulnerable populations in Axshya districts and registered for treatment in RNTCP between March 2016 and February 2017.

**Figure 1.**
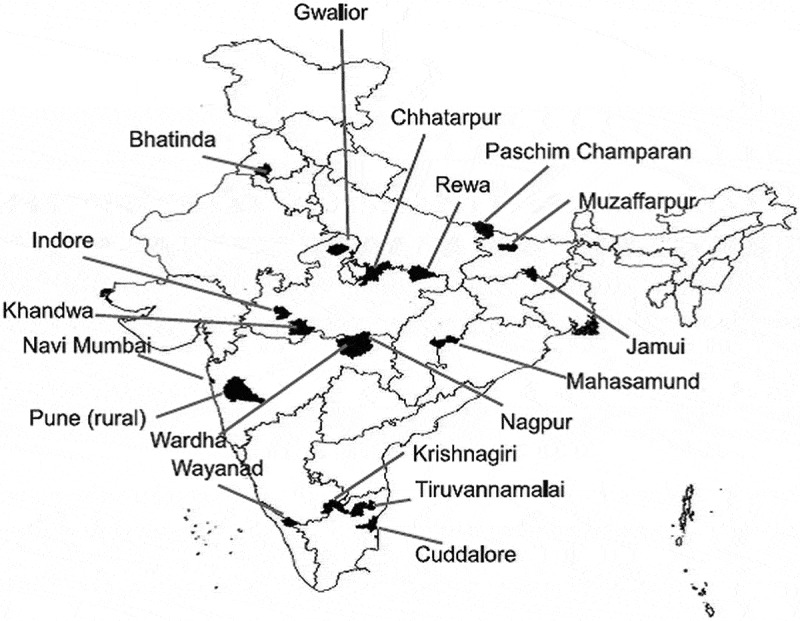
Map of India depicting the randomly sampled Axshya districts (n = 18) under Axshya *SAMVAD* study, India (2016–17) [20]*. *SAMVAD –* sensitization and advocacy in marginalised and vulnerable areas of the districtAxshya *SAMVAD* – an active case finding strategy under project Axshya implemented by The Union South-East Asia (USEA), New Delhi, India, across 285 districts of India* Reprinted from Shewade HD et al. [20] under a CC BY license, with permission from International Union Against Tuberculosis and Lung Disease (The Union), ©The Union 2017

For this study, 18 out of 285 Axshya districts were selected using simple random sampling (Figure 1). These 18 districts belonged to seven states. All people with ACF-diagnosed TB were included. Every month, an equal number of people with PCF-diagnosed TB were included from each Axshya district using simple random sampling (no matching was done). In the last two months of data collection, we observed that the non-response rate for interview was higher among the PCF when compared to the ACF group; hence, we randomly enrolled two PCF-diagnosed people for every ACF-diagnosed person (2:1 ratio). People with mixed/contaminated exposure to ACF were excluded. These were identified through PCF but ACF had been conducted in the village before date of diagnosis [20].

#### Sample size

The ‘Axshya *SAMVAD* study’ was primarily designed to study the differences in total pre-treatment delay among ACF and PCF-diagnosed people with TB, with a sample size of 325 in each group [20–22]. Assessment of the relative difference in TB treatment outcomes was the secondary objective. This sample size of 325 in each group had sufficient power to detect a difference of 9.5% in the proportion of unfavourable treatment outcomes at the end of treatment in the ACF and the PCF groups, assuming a power of 80%, 5% alpha error and a design effect of two (cluster selection of districts).

### Variables, sources and data collection

The data collection form to collect baseline characteristics at treatment registration was divided into two parts: the first part collected through TB treatment register, treatment card and project Axshya records (see Suppl Annex 2) and the second part through structured closed-ended interviews at patient’s residence (see Suppl Annex 3). The interviewers were trained project staff who collected data during their routine supervisory visits. Scanned copies of filled data collection form and audio records of interviews were shared over a cloud platform accessible to investigators. All filled data collection forms were reviewed near real time and randomly sampled audio records were reviewed to ensure data quality from patient interviews [20].

Programme documented treatment outcomes were prospectively tracked at the end of the intensive phase and after one year of registration in the TB treatment register and classified as favourable and unfavourable (Table 1) [26].

**Table 1.** Operational definitions of treatment outcomes for people with TB (not known to be drug-resistant TB) used in India’s national TB programme (2016–17).

Outcome	Definition*
**End of the intensive phase**	
Microbiological conversion	Sputum negative at the end of the intensive phase
Microbiological non-conversion	Sputum positive after extension of one month of the intensive phase
Lost to follow up	Did not start treatment or treatment was interrupted for two consecutive months or more
Died	Dies for any reason before starting or during the course of treatment.
Not evaluated	No follow-up sputum microscopy results are available. This includes people with TB ‘transferred out’ to another treatment unit and the sputum examination results are unknown to the reporting unit
Favourable outcome	Microbiological conversion
Unfavourable outcome*	All outcomes other than microbiological conversion
**End of treatment**	
Cured	People with pulmonary TB who are bacteriologically confirmed TB at the beginning of treatment who was smear- or culture-negative in the last month of treatment and on at least one previous occasion.
Treatment completed	Completed treatment without evidence of failure BUT with no record to show that sputum smear or culture results in the last month of treatment and on at least one previous occasion were negative, either because tests were not done or because results are unavailable.
Treatment failed	Sputum smear or culture is positive at month 5 or later during treatment.
Lost to follow-up	Did not start treatment or treatment was interrupted for two consecutive months or more
Died	Dies for any reason before starting or during the course of treatment.
Not evaluated	No treatment outcome is assigned. This includes people with TB ‘transferred out’ to another treatment unit and the outcome is unknown to the reporting unit.
Favourable outcome	The sum of cured and treatment completed
Unfavourable outcome*	All outcomes other than cured and treatment completed

TB – tuberculosis

*If patient is transferred to drug-resistant-TB care during TB treatment and there is evidence of patient registering in the drug-resistant-TB centre, then patient will be excluded from this drug-susceptible TB cohort. If there is no evidence of patient registering in the drug-resistant-TB centre, then patient will be included in this drug-susceptible cohort and reported as ‘transferred to DR-TB’ and classified under unfavourable outcomes [26].

### Analysis and statistics

Data (but for treatment outcomes that were single entered in Microsoft Excel (Microsoft, Redmond, WA, USA)) were double-entered and validated using EpiData entry software (version 3.1, EpiData Association, Odense, Denmark). Data analysis was done using STATA (version 12.1, copyright 1985–2011 StataCorp LP USA).

Baseline characteristics of the ACF and the PCF groups were compared using chi-square test/independent t-test/Mann Whitney U test, as appropriate. Treatment outcomes were assessed using proportions and 95% confidence intervals (CI).

Association between ACF and unfavourable outcomes at the end of treatment was assessed using relative risks (crude and adjusted) and 95% CI. Log binomial regression was used after adjusting for clustering at the level of districts. A baseline characteristic was considered as confounder if it was associated both with ACF (p < 0.05 or clinically significant difference of at least 5%) and unfavourable outcome (p < 0.2), after ruling out multicollinearity. Variables in the causal pathway like delay before diagnosis and treatment and catastrophic costs due to TB diagnosis were not considered as confounders [21,22,27]. HIV was positive only in one patient and therefore not included.

## Results

### Study participant enrolment and baseline characteristics

A total of 661 participants were enrolled. Of them, 89 did not fit into study participant definition and were excluded. Of 572 enrolled and eligible, 275 belonged to the ACF and 297 to the PCF group. Due to patient non-availability during visit to their residence, interviews were not conducted for 107 (18.7%). When compared to those interviewed (n = 465), those not interviewed (n = 107) were more likely to belong to the PCF group, reside in rural areas and have a sputum grading of 3+ at diagnosis. Hence, we performed adjusted analysis in the 572 cohort as well as the 465 cohort. The 572 cohort included patients that were not interviewed. In the 572 cohort, baseline characteristics were consistently available from record review. In the 465 cohort, baseline characteristics were consistently available both from record review as well as interviews.

The baseline characteristics of both the cohorts, stratified by the ACF and the PCF group are depicted in Tables 2 and 3 (see Suppl Table 1 for distribution of participants by districts).

**Table 2.** Baseline characteristics of people with new smear-positive pulmonary TB enrolled in Axshya *SAMVAD* study (includes all enrolled patients – data from record review available) across 18 randomly sampled districts in India, 2016–17 (N = 572).

		Total		Active case finding		Passive case finding		
		[N = 572]		[N = 275]		[N = 297]		
Variable		n	(%)	n	(%)	n	(%)	p value*
**Socio-demographic characteristics**								
Age in years	15–44	320	(56)	136	(49)	184	(62)	0.015
	45–64	185	(32)	101	(37)	84	(28)	
	≥65	66	(12)	38	(14)	28	(9)	
	Missing	1	(<1)	0	(0)	1	(<1)	
	Mean (SD)	42	(17)	44	(17)	39	(17)	0.002
Gender	Male	377	(66)	178	(65)	199	(67)	0.491
	Female	193	(34)	97	(35)	96	(32)	
	Missing	2	(<1)	0	(0)	2	(1)	
Residence	Urban	82	(14)	23	(8)	59	(20)	<0.001
	Rural	483	(85)	248	(90)	235	(79)	
	Missing	7	(1)	4	(2)	3	(1)	
**Clinical characteristics**								
Sputum grading	3+	92	(16)	39	(14)	53	(18)	0.179
	Scanty/1+/2+	406	(71)	194	(71)	212	(71)	
	Positive not quantified	50	(9)	29	(10)	21	(7)	
	Missing	24	(4)	13	(5)	11	(4)	
Weight in kg	<30	9	(2)	6	(2)	3	(1)	0.498
	30–44.9	232	(40)	117	(43)	115	(39)	
	≥45	109	(19)	50	(18)	59	(20)	
	Missing	222	(39)	102	(37)	120	(40)	
HIV status^	Positive	1	(<1)	0	(0)	1	(<1)	-
	Negative	337	(59)	162	(59)	175	(59)	
	Missing	234	(41)	113	(41)	121	(41)	
DM status	DM	10	(2)	5	(2)	5	(2)	0.949
	Not DM	195	(34)	92	(33)	103	(35)	
	Missing	367	(64)	178	(65)	189	(63)	
**Health system characteristics**								
Distance of residence from DMC in km								
	≤5	152	(26)	58	(21)	94	(32)	0.009
	6–10	170	(30)	90	(33)	80	(27)	
	11–15	129	(23)	58	(21)	71	(24)	
	>15	120	(21)	68	(25)	52	(17)	
	Missing	1	(<1)	1	(<1)	0	(0)	
	Median (IQR)	10	(5,15)	10	(6,15)	10	(4,15)	0.020

Column percentage

TB – tuberculosis; *SAMVAD* – sensitization and advocacy in marginalised and vulnerable areas of the district; SD – standard deviation; HIV – human immunodeficiency virus; DM – diabetes mellitus; DMC – designated microscopy centre; IQR – interquartile range; Axshya *SAMVAD* – an active case finding strategy under project Axshya implemented by The Union South-East Asia, New Delhi, India, across 285 districts of India.

*chi-square test/independent t-test/Mann Whitney U test; ^number with HIV very low (n = 1); hence, p value not calculated.

**Table 3.** Baseline characteristics of people with new smear-positive pulmonary TB enrolled in Axshya *SAMVAD* study (includes enrolled patients whose interview was conducted – data from record review and interviews available) across 18 randomly sampled districts in India, 2016–17 (N = 465).

		Total			Active case finding			Passive case finding		
		[N = 465]			[N = 234]			[N = 231]		
Variable		n	(%)		n	(%)		n	(%)	p value*
**Socio-demographic characteristics**										
Age in years	15–44	251	(54)		111	(47)		140	(61)	0.009
	45–64	163	(35)		91	(39)		72	(31)	
	≥65	50	(11)		32	(14)		18	(8)	
	Missing	1	(<1)		0	(0)		1	(<1)	
	Mean (SD)	42	(17)		44	(17)		40	(17)	0.003
Gender	Male	307	(66)		153	(65)		154	(67)	0.721
	Female	157	(34)		81	(35)		76	(33)	
	Missing	1	(<1)		0	(0)		1	(<1)	-
Residence	Urban	58	(12)		17	(7)		41	(18)	<0.001
	Rural	402	(87)		214	(92)		188	(81)	
	Missing	5	(1)		3	(1)		2	(1)	
Education – patient										
	No formal education	217	(47)		133	(57)		84	(36)	<0.001
	Less than primary	67	(14)		30	(13)		37	(16)	
	Up to secondary	149	(32)		57	(24)		92	(40)	
	Higher secondary and above	30	(7)		13	(6)		17	(7)	
	Missing	2	(<1)		1	(<1)		1	(<1)	
Education – head of household										
	No formal education	228	(49)		133	(57)		95	(41)	0.005
	Less than primary	74	(16)		32	(14)		42	(18)	
	Up to secondary	128	(28)		55	(24)		73	(32)	
	Higher secondary and above	33	(7)		12	(5)		21	(9)	
	Missing	2	(<1)		2	(<1)		0	(0)	
Occupation – patient										
	Unemployed	59	(13)		31	(13)		28	(12)	0.283
	Studying	24	(5)		8	(3)		16	(7)	
	Homemaker	82	(18)		45	(19)		37	(16)	
	Daily wage labour	178	(38)		95	(41)		83	(36)	
	Employed-not daily wage	113	(24)		52	(22)		61	(26)	
	Missing	9	(2)		3	(1)		6	(3)	
Occupation – head of household										
	Unemployed	42	(9)		26	(11)		16	(7)	0.073
	Studying	2	(<1)		1	(<1)		1	(<1)	
	Homemaker	10	(2)		4	(2)		6	(3)	
	Daily wage labour	245	(53)		134	(57)		111	(48)	
	Employed-not daily wage	151	(33)		64	(28)		87	(38)	
	Missing	15	(3)		5	(2)		10	(4)	
Monthly income per capita (USD)**										
	(Median (IQR))	16		(7, 31)	13	(6, 24)		16	(8, 31)	0.014
**Clinical characteristics**										
TB in household ever										
	Yes	116	(25)		54	(23)		62	(27)	0.321
	No	347	(75)		180	(77)		167	(72)	
	Missing	2	(<1)		0	(0)		2	(1)	
TB death in household ever										
	Yes	51	(11)		27	(11)		24	(10)	0.704
	No	413	(89)		207	(89)		206	(89)	
	Missing	1	(<1)		0	(0)		1	(<1)	
History of fever***										
	Yes	350	(75)		170	(73)		180	(78)	0.231
	No	105	(22)		58	(25)		47	(20)	
	Missing	10	(3)		6			4	(2)	
History of weight loss***										
	Yes	340	(73)		159	(68)		181	(78)	0.032
	No	113	(24)		66	(28)		47	(20)	
	Missing	12	(3)		9	(4)		3	(2)	
History of hemoptysis***										
	Yes	119	(26)		60	(25)		59	(26)	0.937
	No	336	(72)		168	(72)		168	(73)	
	Missing	10	(2)		6	(3)		4	(1)	
Current Smoker^										
	Yes	113	(24)		65	(28)		48	(21)	0.122
	No	343	(74)		164	(70)		179	(77)	
	Missing	9	(2)		5	(2)		4	(2)	
Current alcohol intake^										
	Yes	130	(28)		61	(26)		69	(30)	0.419
	No	327	(70)		168	(72)		159	(69)	
	Missing	8	(2)		5	(2)		3	(1)	
Sputum grading	3+	83	(18)		34	(15)		49	(21)	0.068
	Scanty/1+/2+	365	(78)		190	(81)		175	(76)	
	Positive not quantified	17	(4)		10	(4)		7	(3)	
Weight in kg	<30	8	(2)		6	(2)		3	(1)	0.540
	30–44.9	200	(43)		102	(44)		98	(42)	
	≥45	96	(21)		44	(19)		52	(23)	
	Missing	161	(35)		83	(35)		78	(34)	
	Mean (SD)	41	(7)		41	(6)		41	(7)	0.781
HIV status^^	Positive	1	(<1)		0	(0)		1	(<1)	-
	Negative	287	(59)		143	(61)		144	(62)	
	Missing	177	(38)		91	(39)		86	(37)	
DM status	DM	9	(2)		4	(2)		5	(2)	0.784
	Not DM	171	(37)		84	(36)		87	(38)	
	Missing	285	(61)		146	(62)		139	(60)	
**Health system characteristics**										
Distance of residence from DMC in km										
	≤5	118	(25)		50		(21)	68	(29)	0.063
	6–10	144	(31)		80		(34)	64	(28)	
	11–15	107	(23)		49		(21)	58	(25)	
	>15	96	(21)		55		(24)	41	(18)	
	Median (IQR)	10	(5,15)		10		(6, 15)	10	(5, 14)	0.090

Column percentage

TB – tuberculosis; *SAMVAD* – sensitization and advocacy in marginalised and vulnerable areas of the district; SD – standard deviation; USD – US dollar; HIV – human immunodeficiency virus; DM – diabetes mellitus; DMC – designated microscopy centre; IQR – interquartile range.

Axshya *SAMVAD* – an active case finding strategy under project Axshya implemented by The Union South-East Asia, New Delhi, India, across 285 districts of India.

*chi-square test/independent t-test/mann whitney U test; **Pre-TB income, average Indian rupee to USD conversion rate in Jan 2018 (1USD = 63.6 Indian rupees), Indian rupee value used for calculating p value; ***history of fever/significant weight loss/haemoptysis between eligibility for sputum examination and diagnosis; ^ consumption of alcohol/smoke form of tobacco anytime in the month before date of diagnosis; ^^number with HIV very low (n = 1); hence, p value not calculated.

### Treatment outcomes

The treatment outcomes in the 572 cohort are depicted in Tables 4 and 5. The proportion of unfavourable outcomes at the end of the intensive phase were 10.5% (95% CI: 7.5%, 14.7%) in the ACF group and 14.1% (95% CI: 10.6%, 18.6%) in the PCF group (p = 0.239). The proportion of unfavourable outcomes at the end of treatment were 10.2% (95% CI: 7.1%, 14.3%) in the ACF group and 12.5% (95% CI: 9.2%, 16.7%) in the PCF group (p = 0.468). Loss to follow-up was not significantly different (5.8% in ACF versus 7.5% in PCF, p = 0.50).

**Table 4.** Treatment outcomes among people with new smear-positive pulmonary TB enrolled in Axshya *SAMVAD* study across 18 randomly sampled districts in India, 2016–17, stratified by Axshya *SAMVAD* exposure (N = 572).

		Total		Active case finding		Passive case finding	
Outcomes		n	(%)	n	(%)	n	(%)
Total		**572**	**(100)**	**275**	**(100)**	**297**	**(100)**
Treatment outcomes – end of intensive phase							
Favourable							
	**Microbiological conversion**	**501**	**(87.6)**	**246**	**(89.5)**	**255**	**(85.9)**
Unfavourable	**Total**	**71**	**(12.4)**	**29**	**(10.5)**	**42**	**(14.1)**
	Microbiological non-conversion	16	(2.8)	8	(2.9)	8	(2.7)
	Lost to follow up	34	(5.9)	13	(4.6)	21	(7.1)
	Died	10	(1.8)	4	(1.5)	6	(2.0)
	Transfer to DR-TB	0	(0)	0	(0)	0	(0)
	Not evaluated	11	(1.9)	4	(1.5)	7	(2.3)
Treatment outcomes – end of treatment							
Favourable	**Total**	**507**	**(88.7)**	**247**	**(89.8)**	**260**	**(87.5)**
	Cured	434	(75.9)	212	(77.1)	222	(74.7)
	Treatment completed	73	(12.8)	35	(12.7)	38	(12.8)
Unfavourable*	**Total**	**65**	**(11.3)**	**28**	**(10.2)**	**37**	**(12.5)**
	Lost to follow up	38	(6.7)	16	(5.8)	22	(7.5)
	Died	18	(3.1)	7	(2.6)	11	(3.7)
	Treatment Failure	2	(0.3)	2	(0.7)	0	(0)
	Transfer to DR-TB*	2	(0.3)	1	(0.4)	1	(0.3)
	Not evaluated	5	(0.9)	2	(0.7)	3	(1.0)

Column percentage

TB – tuberculosis; *SAMVAD* – sensitization and advocacy in marginalised and vulnerable areas of the district; Axshya *SAMVAD* – an active case finding strategy under project Axshya implemented by The Union South-East Asia, New Delhi, India, across 285 districts of India; DR-TB – drug resistant TB.

*Patient was transferred to drug-resistant-TB care during TB treatment, but there is no evidence of patient registering in the drug-resistant-TB centre, hence patient included in this drug-susceptible TB cohort and reported as ‘transferred to DR-TB’ and classified under unfavourable outcomes [26].

**Table 5.** Effect of Axshya *SAMVAD* on unfavourable outcomes at the end of treatment [26] among people with new smear-positive pulmonary TB enrolled in Axshya *SAMVAD* study [20] across 18 randomly sampled districts in India, 2016–17.

		Total	Outcome					
Cohort*		N	n	(%)	RR	(95% CI)	aRR^^^	(95% CI)
‘572ʹ cohort								
	Axshya *SAMVAD* (ACF)	275	28	(10.2)	0.82	(0.51, 1.30)	0.83	(0.56, 1.21)
	Non-Axshya *SAMVAD* (PCF)	297	37	(12.5)	Ref			
‘465 cohort’								
	Axshya *SAMVAD* (ACF)	234	21	(9.0)	0.86	(0.48, 1.52)	1.05	(0.62, 1.77)
	Non-Axshya *SAMVAD* (PCF)	231	27	(11.7)	Ref			

Row percentage

TB – tuberculosis; *SAMVAD* – sensitization and advocacy in marginalised and vulnerable areas of the district; Axshya *SAMVAD* – an active case finding strategy under project Axshya implemented by The Union South-East Asia, New Delhi, India, across 285 districts of India; DR-TB – drug resistant TB; ACF – active case-finding; PCF – passive case-finding; RR – crude relative risk; aRR – adjusted relative risk.

*The ‘572ʹ cohort includes all patients enrolled in the study and for whom baseline data was available from record review; the ‘465ʹ cohort includes the participants among the 572 cohort for whom baseline data was available from record review as well as from patient interviews.

**^^^**Using log binomial regression after adjusting for clustering at district level; Confounders adjusted in the ‘572ʹ cohort were age, sex and distance of residence from microscopy centre; Confounders adjusted in the ‘465ʹ cohort were age, sex, distance of residence from microscopy centre, history of fever, history of weight loss, type of first health-care provider visited for diagnosis, education and occupation of head of household. A baseline characteristic was considered as confounder if it was associated both with Axshya *SAMVAD* exposure (p < 0.05 or clinically significant difference of at least 5%) and unfavourable outcome (p < 0.2), after ruling out multicollinearity. Variables in the causal pathway were not considered (delay before diagnosis and treatment, catastrophic costs due to TB diagnosis) [21,22,27] HIV was found positive only in one patient and therefore not included.

### Adjusted analysis

In the 572 cohort, after adjusting for age, sex and distance of residence from DMC (see Suppl Table 2), ACF-diagnosed people had 17% lower chance of unfavourable outcomes at the end of treatment when compared to PCF-diagnosed, but this was not statistically significant [aRR: 0.83 (95% CI: 0.56, 1.21)]. (Table 5) In the 465 cohort too, there was no association between ACF and unfavourable outcomes [aRR: 1.05 (95% CI: 0.62, 1.77)] after adjusting for age, sex, distance of residence form DMC, history of fever, history of weight loss, type of first health-care provider visited for diagnosis, education and occupation of head of household (see Suppl Table 3 and Table 5).

## Discussion

In this study from marginalised and vulnerable populations in India, we did not find significant differences in the treatment outcomes of people with new smear-positive pulmonary TB detected through community-based ACF and PCF. The strength of our study was an exhaustive list of baseline variables that were collected from interviews and these were adjusted for. This has been cited as a limitation previously [11].

There were some limitations in our study. First, this study was limited by sample size. The sample size of 650 (325 in each group) was powered to detect a minimum difference of 9.5% in treatment outcomes. In addition, there were some patients who had to be excluded after enrolment as well. Of 661 enrolled, 89 were excluded due to errors in eligibility assessment and for 107, patient interviews could not be done. However, it was consistently seen in both 465 and 572 cohorts (with and without the interview data to adjust for, respectively) that ACF was not associated with unfavourable outcomes. Second, data on diabetes status and weight, which are key confounders and are routinely collected within the programme, were missing for a significant number of people with TB. Third, we were not able to assess and compare the pre-treatment loss to follow-up as we included people that were registered for treatment. Finally, dates were not consistently available for all the outcomes, hence we were not able to compare the timing of outcomes and perform a time to event analysis.

Limitations notwithstanding, the study had some key findings. The findings of the 572 cohort are similar to the Myanmar study (2014–16) [11]. There was a 17% reduction of unfavourable outcomes in our study while a 12% reduction was reported in the Myanmar study, though neither was statistically significant. In both these analysis, factors that were routinely reported within the programme were adjusted for. In our study, not only the 95% CI was insignificant, but also wide. Due to the wide CI, this finding in the 572 cohort that the differences are not statistically significant is inconclusive. However, when we adjusted for the additional variables that were available from interviews in the 465 cohort, the 17% reduction disappeared and the relative risk was almost one (no association). Overall, the 95% CIs were wide either due to limited sample size or multiple and too many variables for adjustment compared to the number of adverse outcomes. Studies with larger sample sizes are urgently required to rule out these extreme differences in treatment outcomes.

We were able to assess the effect of ACF on treatment outcomes at individual level. There have been studies looking at the effect of ACF or ACF as a part of a larger package to improve TB prevention and control, where population-level treatment success rates were compared in the intervention area before and after the intervention (potential confounders were not adjusted for) [9,10]. In these studies, the effect of baseline secular trends on change in treatment outcomes cannot be ruled out. In addition, in a comprehensive TB care package, it is difficult to tease out the effect of community-based ACF [9].

Though the ACF under project Axshya reduced diagnosis delay and prevalence of catastrophic costs due to TB diagnosis, it did not translate into improved treatment outcomes. However, similar to the Myanmar study, the finding in our study allays fears that people with TB detected through ACF could have poor outcomes due to delay in treatment initiation, poor adherence and treatment completion as they are relatively healthier and the diagnosis is not patient-initiated [11]. Additionally, the treatment initiation delays in our cohort of people with TB were not significantly different in the ACF and PCF groups (published elsewhere) [22]. This warrants systematic adherence assessment studies and a qualitative systematic enquiry in India and similar high TB burden countries which can feed into interventions to ensure that early diagnosis through ACF translates into improved outcomes. There is little evidence to support interventions to improve adherence among people with ACF-diagnosed TB from marginalised and vulnerable populations. These risk groups could have additional predictors of unfavourable outcomes [28].

## Conclusion

We did not find significant differences in treatment outcomes of people with TB detected through active case finding when compared to passive case finding in marginalised and vulnerable populations of India. This was despite the fact that the active case finding activity resulted in reduction of diagnosis delay and prevalence of catastrophic costs due to TB diagnosis. Due to the wide CIs, studies with larger sample sizes are urgently required to rule out these extreme differences in treatment outcomes. Studies are also required to understand how to translate the benefits of active case finding to improved TB treatment outcomes. This is essential if India is to END TB by 2025, ten years ahead of the global 2035 targets [7,29].

## Paper context

There is limited evidence on the effect of community-based active case finding for tuberculosis on treatment outcomes when compared to passive case finding. In this study from marginalised and vulnerable populations in India, we did not find significant differences in unfavourable outcomes among active and passive case finding diagnosed patients after adjusting for baseline confounders. Studies are required to understand how to translate the benefits of active case finding to improved treatment outcomes.
